# miRNA-7145-cuedc2 axis controls hematopoiesis through JAK1/STAT3 signaling pathway

**DOI:** 10.1038/s41420-024-01977-6

**Published:** 2024-05-02

**Authors:** Yongsheng Xu, Rui Guo, Tao Huang, Chunming Guo

**Affiliations:** Center for Life Sciences, School of Life Sciences, Yunnan University, Kunming, 650500 China

**Keywords:** Haematopoiesis, Developmental biology

## Abstract

Hematopoiesis ensures tissue oxygenation, and remodeling as well as immune protection in vertebrates. During embryogenesis, hemangioblasts are the source of all blood cells. Gata1a and pu.1 are co-expressed in hemangioblasts before hemangioblasts are differentiated into blood cells. However, the genes that determine the differentiation of hemangioblasts into myeloid or erythroid cell lineages have not been fully uncovered. Here we showed that miRNA-7145, a miRNA with previously unknown function, was enriched in erythrocytes at the definitive wave, but not expressed in myeloid cells. Overexpression and loss-of-function analysis of miRNA-7145 revealed that miRNA-7145 functions as a strong inhibitor for myeloid progenitor cell differentiation while driving erythropoiesis during the primitive wave. Furthermore, we confirmed that cuedc2 is one of miRNA-7145 targeted-genes. Overexpression or knock-down of cuedc2 partially rescues the phenotype caused by miRNA-7145 overexpression or loss-of-function. As well, overexpression and loss-of-function analysis of cuedc2 showed that cuedc2 is required for myelopoiesis at the expense of erythropoiesis. Finally, we found that overexpression of zebrafish cuedc2 in 293 T cell inhibits the JAK1/STAT3 signaling pathway. Collectively, our results uncover a previously unknown miRNA-7145-cuedc2 axis, which regulate hematopoiesis through inhibiting the JAK1/STAT3 signaling pathway.

## Introduction

Hematopoiesis is essential for vertebrate organisms ensuring tissue oxygenation and remodeling as well as immune protection. Blood cells can be classified into leukocytes and erythrocytes by function [1]. The leukocytes constitute the main immunity cells, and include macrophages, mast cells, dendritic cells, neutrophils, basophils, eosinophils, T cells, B cells, and NK cells [2]. They are also involved in tissue remodeling upon embryonic development and organ regeneration [3–5]. The major function of the erythrocytes is to bind and transport O_2_ and CO_2_ [2] throughout the body to support oxidative phosphorylation in the tissues and cells [6]. Together, all these blood cells cooperate with each other in order to maintain physiological homeostasis and act against pathogens. As all blood cells are derived from hematopoietic stem cells (HSCs), it is extremely important to understand the mechanism of HSC production and lineage segregation.

Hematopoiesis is a highly conserved process in vertebrates since the evolution of fish. The process of hematopoiesis in vertebrates can be divided into two main stages: the primitive wave and the definitive wave [2]. The primitive wave mainly produces erythrocytes, as well as macrophages and granulocytes during early embryonic development, to ensure optimal oxygen supply for the rapidly growing embryo [7]. Definitive hematopoiesis later involves HSCs, which are multipotent and can give rise to all blood lineages of the adult organism. In vertebrates, definitive HSCs are born in the aorta-gonad-mesonephros (AGM) region of the developing embryo. They migrate to the fetal liver and then to the bone marrow, which is the location for HSCs in adults [2].

In mammals, hematopoiesis originates in the ventral mesoderm of the embryo, where some cells are specialized into hemangioblasts, which have the ability to differentiate into both endothelial cells and HSCs [8]. In mice, hemangioblasts first appear in the blood island in the yolk sac, and mainly differentiate into primitive erythroid and primitive myeloid cells [9]. The cell fate of the erythroid and myeloid lineage is mainly determined by two transcription factors, Gata1 and Pu.1. These two proteins have a cross-repressive relationship through direct protein interaction [10, 11]. However, there exist many unsolved questions about other co-regulators involved in erythroid and myeloid cell differentiation. Previous studies have shown that Gata1 and Pu.1 are co-expressed before HSC fate decision [12, 13]. Therefore, there may be unknown factors involved in the cell fate decision of HSCs, which can regulate erythropoiesis and myeloid cell differentiation.

Zebrafish have the advantages of in vitro embryonic development, transparent embryos, easy genetic manipulation, and, like mammals, contain all blood cell types [14]. Therefore, zebrafish is an ideal animal to study the regulation of HSC differentiation in vertebrates. The location of hematopoiesis in zebrafish is slightly different from that of mammals [15]. Zebrafish embryonic hemangioblasts first appear in the anterior lateral mesoderm (ALM) and posterior lateral mesoderm (PLM), which together resemble the blood islands in mammalian yolk sacs [1, 16]. Interestingly, hemangioblasts in the PLM mainly differentiate into primitive erythroid, while hemangioblasts in the ALM mainly differentiate into primitive myeloid cells [15]. The molecular mechanism of these lineage determinations remain unknown.

MicroRNAs (miRNAs) are encoded by endogenous genes. When expressed, the messenger RNA (mRNA) undergoes a series of cleavage events to ultimately produce a class of small non-coding single-stranded RNA molecules with a length of about 22 nucleotides [17]. They are widely involved in the regulation of the expression of their targets by controlling both the stability of mRNA and translation at the cellular level [18]. There is growing evidence that miRNAs play an important role in development and intracellular homeostasis by regulating numerous, different biological processes [19–22]. However, there are many miRNAs with unknown functions that remain to be studied in zebrafish.

In the present study, we found that an unknown-function miRNA, miRNA-7145, was enriched in erythrocytes at the definitive wave, but it was not expressed in myeloid cells. Overexpression and knock-down experiments showed that overexpression of miRNA-7145 enhanced erythropoiesis while decreasing myeloid progenitor cell differentiation, in contrast, Knock-down of miRNA-7145 enhanced myeloid progenitor cell differentiation while decreasing erythropoiesis. In addition, the phenotype of blood development caused by miRNA-7145 knock-down can be rescued by miRNA-7145 supplement. Moreover, we found that miRNA-7145 can directly regulate the expression of cuedc2, thereby regulating the hematopoiesis. Overexpression or knock-down of cuedc2 partially rescues the phenotype caused by miRNA-7145 overexpression or loss-of-function, respectively. Similar to the miRNA-7145 knock-down, overexpression of cuedc2 enhanced myeloid progenitor cell differentiation while decreasing erythropoiesis, in contrast, knock-down of cuedc2 inhibited myeloid progenitor cell differentiation. Finally, we found that cuedc2 controls hematopoiesis by inhibiting the JAK1/STAT3 signaling pathway. These results provide a new insight into the regulation of hemangioblast differentiation.

## Results

### miRNA-7145 is enriched in erythrocytes at the definitive wave, but not expressed in myeloid cells

**Fig. 1:**
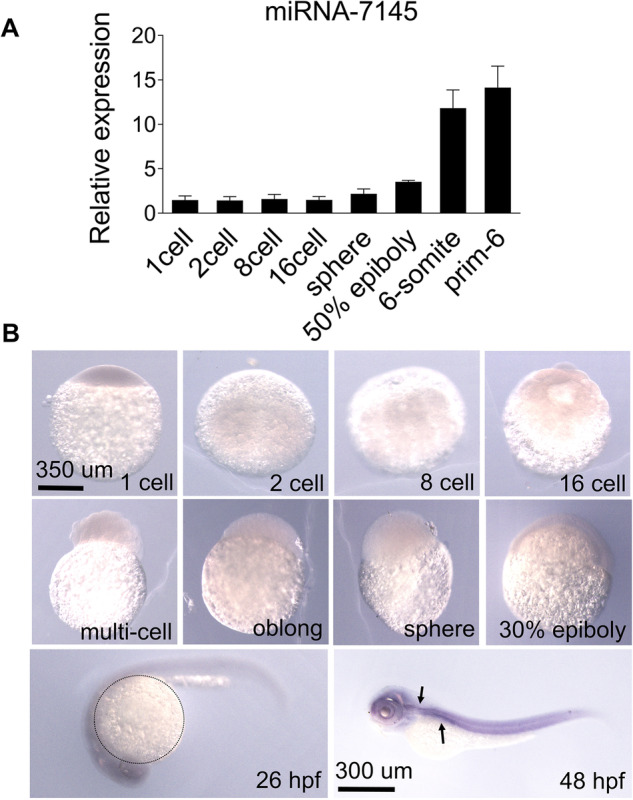
Spatial-temporal expression pattern of miRNA-7145 in wild-type embryos. **A** Quantitative RT-PCR showing miRNA-7145 expression during embryogenesis. The data were calculated with two biological repetitions. Error bars represent SEM. **B** Whole mount in situ RNA hybridization showing the miRNA-7145 expression pattern in wild-type embryos. The arrows indicate the expression site of miRNA-7145. Circle, yolk sac. Scale bar, 1–30% epiboly 350 μm, 26 hpf-48 hpf, 300 μm.

By analyzing newly discovered miRNAs in zebrafish [23], we hypothesized that miRNA-7145 may play an important role in zebrafish embryonic development. Therefore, in order to explore the function of miRNA-7145, qPCR and in situ hybridization were used to detect its expression pattern in zebrafish embryos. During the period from 1 cell to 50% epiboly, miRNA-7145 was detected at extremely low amounts. It only began to be transcribed from the 6-somite stage, with the expression level gradually increasing with the development of the embryo (Fig. 1A). This result indicates that miRNA-7145 is likely to play an important role in zebrafish organogenesis. To explore the specific function of miRNA-7145, we performed in situ hybridization. These results showed that miRNA-7145 was only weakly expressed from the 1 cell stage to the 30% epiboly period, and began to show a strong expression signal after 24 hpf. However, the signal was not detected in the yolk sac (Fig. 1B). After 48 hpf, the expression pattern of miRNA-7145 was similar to gata1a (Supplemental Fig. 1), which is mainly expressed in erythrocytes (Fig. 1B). These results imply that miRNA-7145 may be essential for erythropoiesis.

### Overexpression of miR-7145 enhances erythropoiesis while decreasing myeloid progenitor cell differentiation

**Fig. 2:**
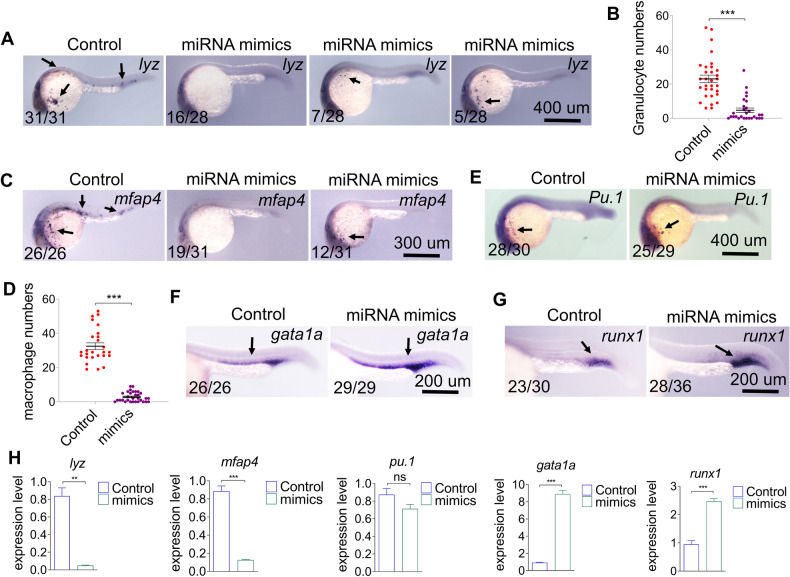
Effect of miRNA-7145 overexpression on hematopoiesis. The embryos were injected with 200 pg miRNA-7145 mimics at the 1 cell stage, and the embryos were collected at 26 hpf. **A**–**E** Detection of myeloid cell marker expression at 26 hpf with whole mount in situ hybridization. **A** *lyz* expression, **C** *mfap4* expression, **E** *PU.1* expression. **B** Statistics of *lyz* positive cells and **D** Statistics of *mfap4* positive cells. **F** *gata1a* expression, **G** *Runx1* expression at 26 hpf. **H** qRT-PCR showing relative RNA expression in control and in embryos injected with miRNA mimics. Relative expression is normalized to β-actin. Data are presented as mean ± SEM. Scale bar is shown in the Figure. *P < 0.05, **P < 0.01, ***P < 0.001.

We found that miRNA-7145 is enriched in erythrocytes, therefore, in order to explore the role of miRNA-7145 in hematopoiesis, we synthesized miRNA-7145 in vitro and detected the phenotype of blood development through overexpression. *Lyz* and *mfap4* are markers of granulocytes and macrophages, respectively [24, 25]. In situ hybridization results showed that overexpression of miRNA-7145 resulted in a dramatic reduction of the numbers of granulocytes and macrophages (Fig. 2A–D). In order to distinguish whether this dramatic decrease was caused by a decrease in the number of myeloid precursor cells or in the failure of myeloid precursor cells to differentiate, we performed in situ hybridization using marker *Pu.1*, which is specific for myeloid precursor cells. Interestingly, overexpression of miRNA-7145 did not affect the number of myeloid precursor cells (Fig. 2E). Therefore, overexpression of miRNA-7145 affected the myeloid progenitor cells differentiation. As the expression patterns of miRNA-7145 and gata1a were similar, we examined the effect of overexpression of miRNA-7145 on erythropoiesis. Overexpression of miRNA-7145 can increase the number of erythrocytes (Fig. 2F). Showing that miRNA-7145 enhances erythropoiesis. Next, we wanted to examine whether overexpression of miRNA-7145 would have an effect on HSCs. Runx1 is a HSCs marker [26]. Similar to erythropoiesis, overexpression also increased HSCs numbers (Fig. 2G). In order to confirm the above results, we performed qPCR of these markers and obtained the same trend as seen in the above experiments (Fig. 2H). Finally, we also did experiments with different doses of miRNA mimics. The results showed that with the increase of the dose of the miRNA mimics, the number of granulocytes and macrophages gradually decreased, while the number of erythrocytes increased at the same time (Supplemental Fig. 2A–E).

### Knock-down of miR-7145 enhances myeloid progenitor cell differentiation while decreasing erythropoiesis

**Fig. 3:**
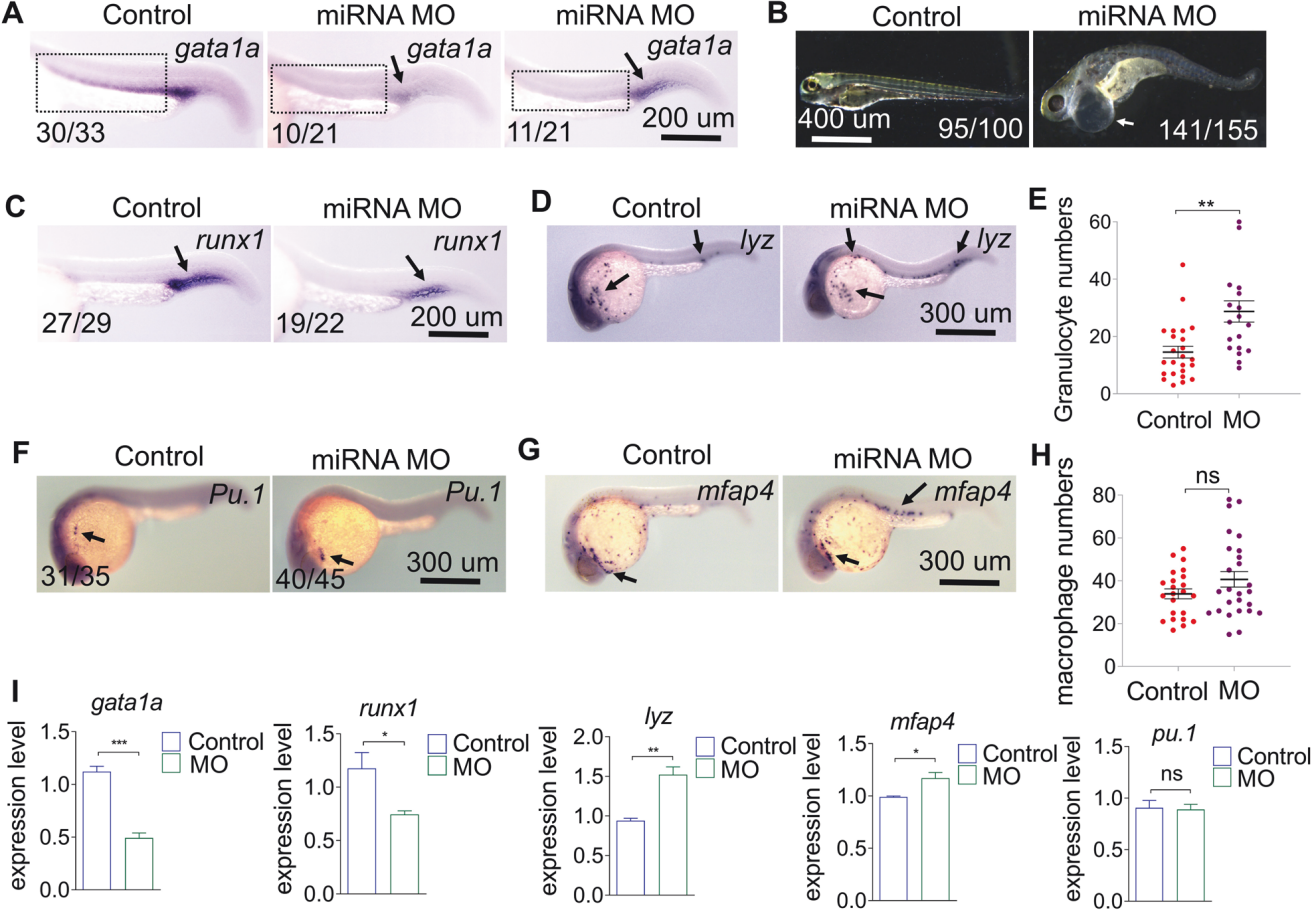
Effect of miRNA-7145 knock-down on hematopoiesis. Embryos were injected with 10 ng miRNA-7145 MO at the 1 cell stage, and the embryos were collected at 26 hpf and 4 dpf. **A** *gata1a* expression, **B** the cardiac edema phenotype occurs 4 dpf after miRNA MO injection, **C** *Runx1* expression. **D**–**H** Detection of myeloid cell marker expression at 26 hpf with whole mount in situ hybridization. **D** *lyz* expression, **F** *Pu.1* expression, **G** *mfap4* expression. **E** Statistics of *lyz*-positive cells and **H** statistics of *mfap4* positive cells. **I** qRT-PCR showing relative RNA expression in control and embryos injected miRNA MO. Relative expression is normalized to β-actin. Data are presented as mean ± SEM. Scale bar is shown in the Figure. *P < 0.05, **P < 0.01, ***P < 0.001.

The miRNA-7145 overexpression experiments showed that miRNA-7145 enhances erythropoiesis. Therefore, we used morpholino oligonucleotides (MO) to knock-down the expression of miRNA-7145 to detect the blood phenotype. Knock-down of miRNA-7145 resulted in a dramatic reduction in the number of erythrocytes (Fig. 3A) and was associated with a cardiac edema phenotype at 4 dpf (Fig. 3B). Similar to erythropoiesis, knock-down of miRNA-7145 also decreased HSCs numbers (Fig. 3C). Knock-down of miRNA-7145 did not affect the number of myeloid progenitor cells (Fig. 3F) and macrophages (Fig. 3G, H), but did cause a slight increase in the number of granulocytes (Fig. 3E). In order to confirm the above results, we performed qPCR of these markers and obtained the same trend as the above experiments (Fig. 3I). Interestingly, although there was no significant increase in the number of macrophages, qPCR showed a slight but significant up-regulation of *mfap4* expression (Fig. 3I). Finally, we also did experiments with different doses of miRNA MO. In contrast to the overexpression of miRNA-7145, the number of erythrocytes gradually decreased with the increase of the miRNA MO dose (Supplemental Fig. 2F).

### The phenotype of blood development caused by miRNA-7145 knock-down can be rescued by miRNA-7145 supplement

In order to avoid the blood phenotype caused by miRNA-7145 MO due to off-target MO, we injected miRNA-7145 mimics and MO together. In situ hybridization results showed that granulocytes and macrophages numbers in co-injection miRNA-7145 mimics and MO embryos were similar to the control group (Supplemental Fig. 3A–D). As well, no significant difference was found in the expression of gata1a in co-injected miRNA-7145 mimics and MO embryos compared with the control group (Supplemental Fig. 3E).

### miRNA-7145 could directly regulate cuedc2 expression

**Fig. 4:**
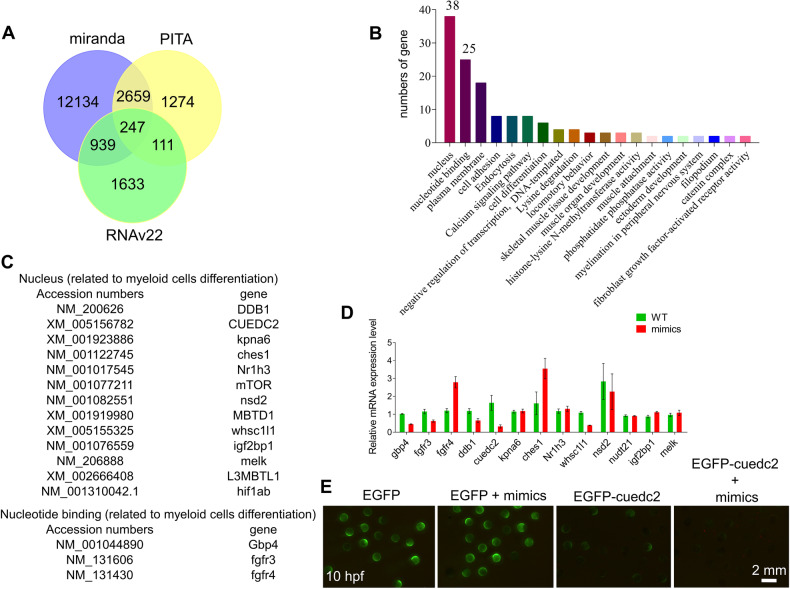
A target gene of miRNA-7145 was identified by bioinformatics analysis and fluorescence reporting experiments. **A** Venn diagram of predicted miRNA-7145 targeted-genes using three different methods. **B** KEGG enrichment analysis of common targeted-genes predicted by the three different methods. **C** List of targeted-genes located in the nucleus and associated with myeloid cells. **D** qRT-PCR showing RNA expression in control embryos injected with miRNA-7145 mimics. The embryos were injected with 200 pg miRNA-7145 mimics at the 1 cell stage, and the embryos were collected at 26 hpf. Relative expression is normalized to β-actin. Data are presented as mean ± SEM. **E** Expression of EGFP and cuedc2 fusion. All components were injected with 200 pg RNA at the 1 cell stage. Scale bar, 2 mm. *P < 0.05, **P < 0.01, ***P < 0.001.

To explore the molecular mechanism of miRNA-7145, we used bioinformatics to find miRNA-7145 targeted-genes. We used three different methods to predict the target genes, and then selected the common target genes of the three methods to apply GO/KEGG enrichment analysis. Through bioinformatics, we predicted 247 genes that are likely to be miRNA-7145 targeted-genes (Fig. 4A), and most of these target genes are located in the nucleus (Fig. 4B). Next, we analyzed these nuclear localized genes and found that many target genes were involved in myeloid cell differentiation (Fig. 4C). In order to verify which genes are truly involved in the regulatory pathway of miRNA-7145, we performed a qPCR analysis after overexpression of miRNA-7145. We found that the expression of five target genes, *Gbp4*, *fgfr3*, *ddb1*, *cuedc2*, and *nsd3*, were down-regulated (Fig. 4D), which meant that these five target genes were likely to be the direct targets of miRNA-7145. Other genes tested: *kpna6*, *Nr1h3*, *nudt21*, *igf2bp1*, *melk*, *nsd2* showed almost no expression change. Interestingly, the expression of two genes, *fgfr4* and *ches1*, was up-regulated. There are two sites on the full-length mRNA sequence of cuedc2 that perfectly bind to the seed sequence of miRNA-7145. Therefore, we fused the full-length mRNA of *cuedc2* with EGFP, then co-injected EGFP-cuedc2 mRNA and miRNA mimics into zebrafish embryos. We found that the fluorescence intensity of embryos injected with EGFP-cuedc2 was lower than that of embryos injected only with EGFP. The fluorescence intensity of embryos co-injected with EGFP-cuedc2 and miRNA mimics was lower than that of embryos injected with EGFP-cuedc2 alone (Fig. 4E). This result showed that the miRNA could directly regulate cuedc2 expression. This indicates that miRNA-7145 is likely to regulate the hematopoiesis process by targeting cuedc2. We also conducted interaction experiments between gbp4 and the miRNA, with the results showing that miRNA-7145 and gbp4 could not directly interact, and did not directly affect the expression of EGFP-gbp4 (Supplemental Fig. 4).

### Overexpression of cuedc2 can partially rescue the phenotype caused by miRNA-7145 overexpression

As we found that miRNA-7145 can directly target cuedc2 expression, we hypothesized that miRNA-7145 may directly regulate the hematopoiesis process through cuedc2. In order to detect that the blood phenotype caused by the overexpression of miRNA-7145 is caused by down-regulation of cuedc2 expression, we co-injected cuedc2 mRNA and miRNA-7145 in embryos to detect erythropoiesis and myeloid progenitor cell differentiation. We found that the number of macrophages in the embryos co-injected with miRNA and cuedc2 was significantly higher than in the embryos injected with miRNA alone, but slightly lower than in the control group (Supplemental Fig. 5A, B). There was no significant difference in the number of granulocytes in embryos co-injected with miRNA and cuedc2 compared to embryos injected with miRNA alone (Supplemental Fig. 5C, D). The expression of gata1a in embryos injected with miRNA mimics was higher than that in the control group, while the expression of gata1a in embryos co-injected with miRNA mimics and cuedc2 was similar to that in the control group (Supplemental Fig. 5E).

### Knock-down of cuedc2 can rescue the phenotype caused by miRNA-7145 knock-down

Similarly, knock-down of miRNA-7145 leads to up-regulation of cuedc2 expression. To test whether the blood phenotype caused by knock-down of miRNA-7145 is due to up-regulation of cuedc2 expression, we co-injected miRNA MO and cuedc2 MO into zebrafish embryos to detect erythropoiesis and myeloid progenitor cell differentiation. We found that there was no significant difference in the number of granulocytes in embryos co-injected with miRNA MO and cuedc2 MO compared to the control group. However, the number of granulocytes in the embryos injected with miRNA MO was significantly higher than in the control group (Supplemental Fig. 6A, B). The expression of gata1a in embryos injected with miRNA MO was lower than that in the control group, while the expression of gata1a in embryos co-injected with miRNA MO and cuedc2 MO was similar to that in the control group (Supplemental Fig. 6C).

### Cuedc2 promotes myeloid cell differentiation at the expense of erythropoiesis

**Fig. 5:**
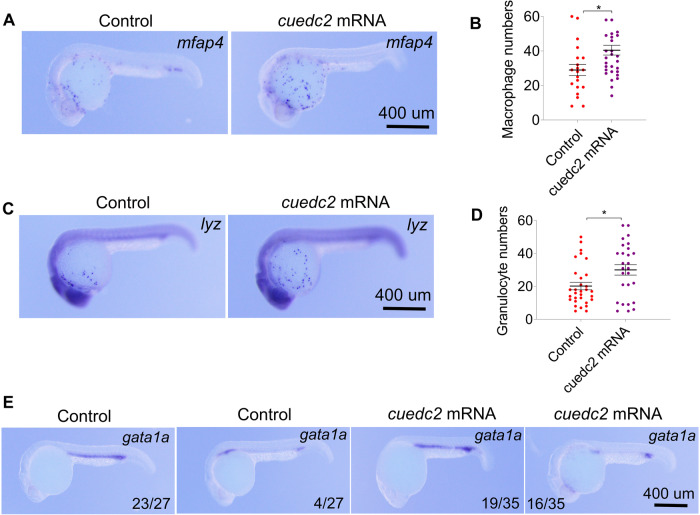
Effect of cuedc2 overexpression on hematopoiesis. Embryos were injected with 200 pg cuedc2 mRNA at the 1 cell stage, and the embryos were collected at 26 hpf. **A**–**D** Detection of myeloid cell marker expression at 26 hpf with whole mount in situ hybridization. **A** *mfap4* expression, **C** *lyz* expression. **B** Statistics of *mfap4* positive cells and **D** statistics of *lyz* positive cells. **E** *gata1a* expression. Scale bar, 400 μm. *P < 0.05, **P < 0.01, ***P < 0.001.

**Fig. 6:**
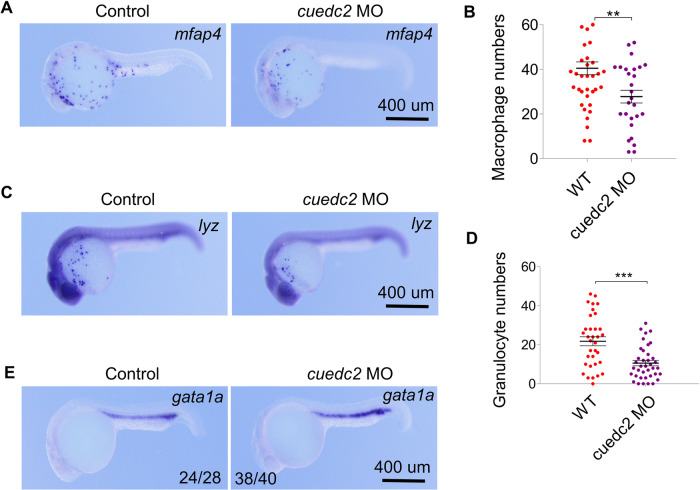
Effect of cuedc2 knock-down on hematopoiesis. The embryos were injected with 10 ng cuedc2 MO at the 1 cell stage, and the embryos were collected at 26 hpf. **A**–**D** Detection of myeloid cell marker expression at 26 hpf with whole mount in situ hybridization. **A** *mfap4* expression, **C** *lyz* expression. **B** Statistics of *mfap4* positive cells and **D** statistics of *lyz* positive cells. **E** *gata1a* expression. Scale bar, 400 μm. *P < 0.05, **P < 0.01, ***P < 0.001.

The previous data showed that miRNA-7145 regulates hematopoiesis by regulating the expression of cuedc2, therefore cuedc2 should regulate hematopoiesis. To test this, we overexpressed cuedc2 in embryos and analyzed blood development. Similar to miRNA-7145 knock-down, overexpression of cuedc2 increases granulocytes and macrophages numbers compared with the control group (Fig. 5A–D). Similarly, overexpression of cuedc2 inhibits erythropoiesis (Fig. 5E). In contrast to cuedc2 overexpression, knock-down of cuedc2 results in a decrease in the number of macrophages and granulocytes (Fig. 6A–D). Cuedc2 knockdown also promotes erythropoiesis (Fig. 6E).

### Zebrafish cuedc2 inhibits the human JAK1/STAT3 signaling pathway

**Fig. 7:**
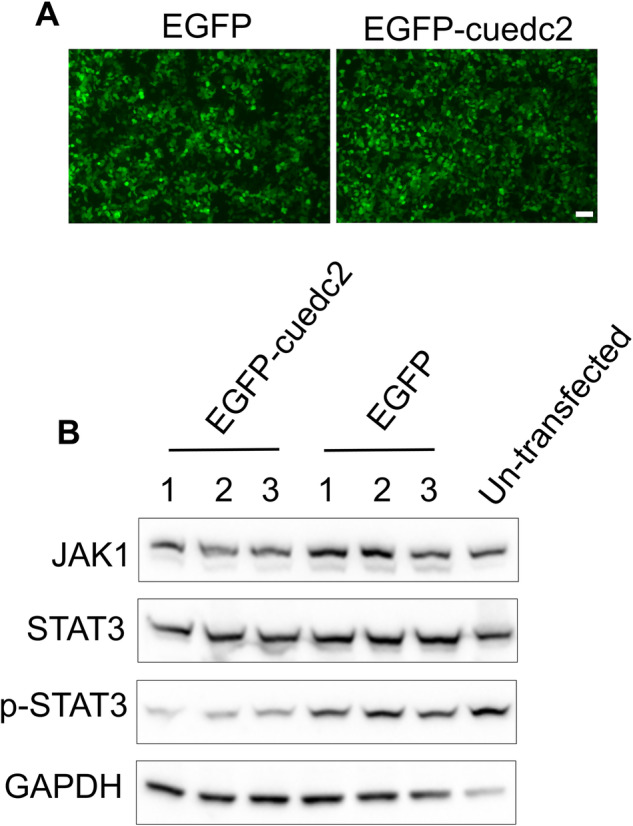
Effect of zebrafish cuedc2 overexpression on the JAK1/STAT3 signaling pathway. **A** 293 T cells were transfected with EGFP plasmid or EGFP-cuedc2 plasmid, and green fluorescence was captured with a fluorescence microscope. **B** 293 T cells were collected to performed western blot. Numbers indicate biological replicates. Scale bar, 150 μm.

Overexpression of JAK in zebrafish embryos increases the expression of gata1 and hemoglobin, suggesting that JAK plays a crucial role in erythropoiesis in zebrafish [27]. Many studies have shown that the JAK/STAT signaling pathway plays an important role in zebrafish hematopoiesis [28–30]. It has been reported that cuedc2 can interact with SOCS1, and SOCS1 can inhibit the JAK1/SATA3 signaling pathway [31]. Therefore, we speculated that cuedc2 is likely to regulate erythropoiesis by inhibiting the JAK1/STAT3 signaling pathway. To verify this relationship, we overexpressed zebrafish cuedc2 in 293 T cells and then detected the changes in the JAK1/STAT3 signaling pathway. The results showed that overexpression of zebrafish cuedc2 inhibited the expression of JAK1 and phosphorylation of STAT3 in 293 T cells (Fig. 7).

## Discussion

Cell fates are mainly determined by transcription factors, and transcription factors are regulated by post-transcriptional regulation [32]. microRNA (miRNA) is a class of small non-coding RNA of about 22 nt, which is widely involved in the post-transcriptional regulation of genes [33]. So far, it has been reported that miRNAs that affect myeloid cell differentiation includes miR-233 [34], miR-146a [35], miR-21 [36], miR-196 [37] and miR-142-3p [38]. It is well known that miR-144 and miR-451 are essential for erythrocyte proliferation and differentiation [39, 40]. More and more evidence have shown that miRNA is involved in the hematopoiesis, but so far, it remains to be shown that miRNA can regulate both erythropoiesis and myeloid cell differentiation. Here, we discovered that a novel miRNA-7145-cuedc2 axis controls hematopoiesis through the JAK1/STAT3 signaling pathway (Supplemental Fig. 7).

Hematopoiesis in zebrafish embryos mainly occurs in two waves, which is are termed the primitive wave and definitive wave [1, 41]. The primitive wave mainly produces erythrocytes and limited myeloid cells [15]. Macrophages and granulocytes are specified from common myeloid progenitor cells in the primitive wave. Around 11 hours post fertilization, the myeloid progenitor cells are derived from the ALM, while erythrocytes are mainly derived from the PLM [42]. Around 30 hours post fertilization, the transient definitive wave arises in the posterior blood island (PBI), which can produce erythromyeloid progenitors (EMPs) with the ability to differentiate into both erythrocytes and myeloid cells. Subsequently, the embryo enters the definitive wave, and the ventral wall of the dorsal aorta produces definitive HSCs, which then migrate to the posterior region of the tail, called the caudal hematopoietic tissue [42]. The expression pattern of miRNA-144 and miRNA-451 are similar to gata1a in the primitive wave, and they are expressed in the intermediate cell mass (ICM) at 24 hpf [40]. However, miRNA-7145 is expressed in all tissues at 24 hpf, but not in the yolk sac. It is possible that miRNA-7145 may have other functions in addition to its hematopoietic functions during early embryonic development. Interestingly, in the definitive wave, the expression pattern of miRNA-7145 is similar to gata1a at 48 hpf. This suggests that miRNA-7145 may be a key determinant of HSCs lineage specification.

Knock-down of miRNA-451 could increase the number of immature erythroid cells in zebrafish [40]. However, knock-down of miR-7145 enhanced myeloid progenitor cell differentiation at the expense of erythropoiesis. This result is in contrast to the overexpression of miRNA-7145, suggesting that the phenotype caused by miRNA-7145 knock-down or overexpression is not due to off-target effects. To test this, we also did co-injection of miRNA mimics and MO, and the blood phenotype disappeared as hypothesized.

A target gene of miRNA-451, gata2, was identified by fluorescence reporting experiments [40]. Similarly, we also identified cuedc2, a target gene of miRNA-7145, through bioinformatics analysis and fluorescence reporting experiments. Overexpression of cuedc2 can partially rescue the phenotype caused by overexpression of miRNA-7145. Cuedc2, a significant inflammation-related factor [43], was highly expressed in peritoneal macrophages, and the expression level was dramatically increased when monocytes differentiate into macrophages [44]. However, it is unclear whether cuedc2 is involved in hematopoiesis. Here we showed that miRNA-7145 controls hematopoiesis through regulating cuedc2 expression. Moreover, we found that cuedc2 enhanced myeloid progenitor cell differentiation at the expense of erythropoiesis. The role of the EPO-JAK-STAT pathway in erythropoiesis is highly conserved in vertebrates. In zebrafish, erythropoietin (EPO) is required for erythropoiesis in both the primitive and definitive wave [45]. Once EPO binds to the EPO-receptor, it recruits JAK, a tyrosine kinase that phosphorylates the EPO-receptor. In zebrafish, overexpression of the JAK/STAT3 signaling pathway promotes erythropoiesis [27–30]. Here, we found that zebrafish cuedc2 can inhibit the human JAK1/STAT3 signaling pathway.

In conclusion, we found that miRNA-7145-cuedc2 axis controls hematopoiesis through the JAK1/STAT3 signaling pathway. This study not only provides new insight into erythropoiesis and myeloid cell differentiation, but also provides a possible evolutionary conserved mechanism for the regulation of the differentiation of HSCs.

## Materials and methods

### Maintenance of zebrafish

Zebrafish husbandry and embryo manipulations were performed as described [46]. The animal protocols have been approved by the Internal Review Board of Yunnan University.

### Microinjection of embryos

Microinjection of embryos were performed as previously reported [47].

### Overexpression and knock-down of miRNA-7145

Briefly, miRNA-7145 mimics were synthesized by Beijing Tsingke Biotech Co., and the miRNA-7145 morpholino was synthesized by gene tools. The miRNA-7145 MO sequence is TGGTAACCATTGGCTTCCATTGTTG. miRNA-7145 mimics and miRNA-7145 MO were dissolved in Rnase-free water. 200 pg miRNA-7145 mimics or 10 ng miRNA-7145 MO were injected into 1 cell embryos.

### RNA extraction and reverse transcription

RNA was extracted with Trizol reagent (15596026, Invitrogen™). Briefly, 1 ml of trizol was added to the embryo and ground. Next 200 μl chloroform was added, the mixture was allowed to set at room temperature for 5 minutes, and then centrifuged at 4 °C. 300 μl of supernatant was taken to a new tube. 300 μl isopropyl alcohol was then added to the supernatant, it was centrifuged at 4 °C, the supernatant was removed and 700 μl of 75% ethanol was added, followed by centrifugation at 4 °C. The ethanol was next discarded and 20 μl of water was added to dissolve the RNA. RNA reverse transcription was conducted using PrimeScript™ RT reagent Kit (RR037Q, Takara). For miRNA qPCR, RNA reverse transcription was conducted using the miRcute Plus miRNA First-strand cDNA Kit (KR211, TIANGEN).

### Real-time quantitative polymerase chain reaction (qPCR)

qPCR was performed using PowerUp™ SYBR™ Green Master Mix (A25741, ThermoFisher). For miRNA qPCR, the miRcute Plus miRNA qPCR Kit (fp411, TIANGEN) was used. The qPCR procedure followed the kit manufacturer’s instructions. The qPCR primers used are shown in Supplemental Table 1. Data were normalized to the level of β-actin and quantified using the delta/delta −Ct method.

### Whole mount in situ hybridization

Whole mount in situ hybridization (WISH) was performed as previously reported [48]. Briefly, digoxigenin-labeled RNA probes were synthesized using a T7 in vitro transcription system (2540 A, Takara). The primers used to synthesize probes is shown in Supplemental Table 2. Embryos were fixed in 4% paraformaldehyde (4% PFA) overnight, then sequentially treated with different concentrations (from low to high) of methanol. On the third day, embryos were sequentially treated with different concentrations (from high to low) of methanol. Rehydrated embryos were hybridized with digoxigenin-labeled RNA probe. Embryos were stained using an anti-digoxigenin antibody (11093274910, Roche) and BM purple substrate (11442074001, Roche). Embryos were photographed with a Leica stereomicroscope (Model M156FC).

### EGFP-cuedc2 p construction and EGFP-cuedc2 mRNA microinjection

First, RNA of zebrafish embryos was extracted, and then cDNA was obtained by reverse transcription. The cuedc2 CDS-3’UTR sequence was then cloned from the cDNA. The cloned cuedc2 CDS-3’UTR sequence is shown in Supplemental Fig. 8. The pcs2 plasmid was cut with BamHI restriction enzyme, and the linearized plasmid was recovered using the DNA recovery Kit (NA1111, Sigma-Aldrich). Finally, pcs2, EGFP and cuedc2 CDS-3’ UTR were ligated with the ClonExpress II One Step Cloning Kit (C112, Vazyme). The plasmid were sequenced by Beijing Tsingke Biotech Co.. EGFP-cuedc2 mRNA was obtained by in vitro transcription. 200 pg EGFP-cuedc2 mRNA or 200 pg EGFP-cuedc2 and 200 pg miRNA mimics were injected into the 1 cell stage embryo of zebrafish.

### In vitro transcription

In vitro transcription was performed using mMESSAGE mMACHINE™ SP6 kit (AM1340, Invitrogen™). The in vitro transcription followed the kit manufacturer’s instructions.

### miRNA targeted gene prediction

We used miRanda (default parameter, version: v1.1), PITA (default parameter, select ^△△^G less than or equal to −10 kcal/mol mRNA, version: v6), and RNA22 (default parameter, version: V2) for predicting the target genes of microRNAs, and we used DAVID (https://david.ncifcrf.gov/) to do GO and KEGG enrichment analysis for the predicted target genes.

### Cell transfection and western blot

800 ng of plasmid was transfected into 293 T cells with Lipofectamine™ 2000 (11668030, ThermoFisher), and samples were collected 48 hours after transfection. The collected cells were lysed with RIPA buffer (89901, ThermoFisher) at 4 °C, then centrifuged at 12,000 rpm at 4 °C for 5 min, to obtain the supernatant for western blotting. 10 μL of protein was mixed with 10 μL 2x loading buffer and boiled at 100 °C for 10 min. The western blot was performed with previously reported method [49]. The antibodies used in this study is as follows: JAK1 primary antibody (ab133666, abcam), STAT3 primary antibody (ab68153, abcam), p-STAT3 primary antibody (ab267373, abcam), and GAPDH (ab8245, abcam).

### Measurements and statistics

All measurements in different experiments were taken from distinct samples. Data analysis was performed with unpaired *t* test using Graphpad prism software.

### Supplementary information

Supplemental Data

Supplemental Figure 1

Supplemental Figure 2

Supplemental Figure 3

Supplemental Figure 4

Supplemental Figure 5

Supplemental Figure 6

Supplemental Figure 7

Supplemental Figure 8

WB original data

## Data Availability

The authors confirm that the data supporting the findings of this study are available within the paper.
