# Supplementary material for: miRNA-7145-cuedc2 axis controls hematopoiesis through JAK1/STAT3 signaling pathway
Source: Cell Death Discov. 2024 May 2;10:209. doi: 10.1038/s41420-024-01977-6 (PMC11066045; doi:10.1038/s41420-024-01977-6)
Supplement: Supplementary file 1 — Supplemental Data [file 41420_2024_1977_MOESM1_ESM.docx]

| primers used for qPCR | |
| --- | --- |
| gata1a-qPCR-F | CACGGGACCATAACGAAGC |
| gata1a-qPCR-R | GCACATACAATAAGCCATACAATCA |
| lyz-qPCR-F | GACCCACCGAGTGCGTTCA |
| lyz-qPCR-R | TTACCCAGCGGGACATCTTA |
| runx1-qPCR-F | GCCGCTCTGGTGGGCAAACT |
| runx1-qPCR-R | GAAGGCGATGGGCAGGGTCT |
| pu1-qPCR-F | ATCACATCCCTCTAACCAATCC |
| pu1-qPCR-F | TTTCGCAGAAGGTCAAGC |
| mfap4-qPCR-F | GGACGGTGATTCAGAGGAGG |
| mfap4-qPCR-R | TCACATTCACAACCCACAGAG |
| β-actin-F | CCCAGACATCAGGGAGTGAT |
| β-actin-R | TCTCTGTTGGCTTTGGGATT |
| ddb1-qPCR-F | AGCGTGAGGCAACGGTGGA |
| ddb1-qPCR-R | ACAGAAACATCACAATGAGGACGACT |
| cuedc2-qPCR-F | AAAACCAGCCCGCAAAT |
| cuedc2-qPCR-R | AGACATCAACCCAAACAACAA |
| kpna6-qPCR-F | TACCATAACCGTCTTCATTTCA |
| kpna6-qPCR-R | CACCACGAACATCCTACAACTA |
| ches1-qPCR-F | GGTGACTTTCTGACACGGGCTAA |
| ches1-qPCR-R | CTCCTTCATCTCCTCGTCCTCCT |
| nr1h3-qPCR-F | GACGCCCTCCACTCCTACA |
| nr1h3-qPCR-R | TCCACCCACGTTTCCCT |
| nsd2-qPCR-F | CGCACAACTACCACCACCC |
| nsd2-qPCR-R | AGTCTGTCAGCCCTGTCATCC |
| whsc1l1-qPCR-F | GCCTGTGCTGTAAAGTGGAA |
| whsc1l1-qPCR-R | AAGTGACAAACATACTGGTGGTG |
| igf2bp1-qPCR-F | TTTGTCGCACGGACTGTTA |
| igf2bp1-qPCR-R | TTGAGGTAGCGTTCCTAGTTATG |
| melk-qPCR-F | AATGACAACGCCCAACCA |
| melk-qPCR-R | TCTGCTGCTGTTCCACCTCT |
| gbp4-qPCR-F | TTACAGCAAGTCCGAGATGAGC |
| gbp4-qPCR-R | CGGGTTACATTAGTCTGGGTGA |
| fgfr3-qPCR-F | AACCATTCCGTGCCTACC |
| fgfr3-qPCR-R | GACTCTTATCCGAACTGTGCC |
| fgfr4-qPCR-F | TGCCTGGCTCACAGTCTTATC |
| fgfr4-qPCR-R | GCAGAGGACCACAATCACAAT |
| Nudt21-qPCR-F | AGGAGTAAATCAGTTCGGCAATA |
| Nudt21-qPCR-R | TCCAGGGAGTTTGAAGAAGGT |

| primers used for WISH | |
| --- | --- |
| gata1a-F | CCTCTGAGCCTTCTCGTTGG |
| gata1a-T7-R | taatacgactcactataggTGGAGCCTGGGACTGTCTTT |
| lyz-F | AAGAATGAAGGGCTTGATGGA |
| lyz-T7-R | taatacgactcactataggGCTCGGAGGCTTTGTTTGC |
| runx1-F | CGCTCTAACTTTCTAAAGGATACGC |
| runx1-T7-R | taatacgactcactataggCATGGCACTTCGCCTCAACT |
| pu1-F | ACATCATCCCACCCAAAGAA |
| PU1-T7-R | taatacgactcactataggTTTCGCAGAAGGTCAAGCAG |
| mfap4-F | ATGGCAATCGTGCTGTTCT |
| mfap4-T7-R | taatacgactcactataggCCAATGGCATGATGGGTG |

**Supplemental Figures**

**Supplemental Figure 1. *Gata1a* expression pattern at 48 hpf.** *gata1a* expression detected by WISH.

**Supplemental Figure 2. Over-expression and knock-down of miRNA-7145 have dose-dependent effects on hematopoiesis.** (A-E) The embryos were injected with different dosages of miRNA mimics at the 1 cell stage, and the embryos were collected at 26 hpf. (A-D) Detection of myeloid cell marker expression at 26 hpf with whole mount *in situ* hybridization. (A) *lyz* expression, (C) *mfap4* expression. (B) Statistics of *lyz* positive cells and (D) Statistics of *mfap4* positive cells. (E) *gata1a* expression. (F) The embryos were injected with different dosages of miRNA MO at the 1 cell stage, and *gata1a* expression was detected at 26 hpf by whole mount *in situ* hybridization. Scale bar is shown in the Figure.

**Supplemental Figure 3. Co-injection of miRNA mimics and MO rescues the blood phenotype.** The embryos were co-injected with 10 ng miRNA-7145 MO and 200 pg miRNA mimics at the 1 cell stage, and the embryos were collected at 26 hpf. (A-D) Detection of myeloid cell marker expression at 26 hpf with whole mount *in situ* hybridization. (A) *mfap4* expression, (C) *lyz* expression. (B) Statistics of *mfap4* positive cells and (D) Statistics of *lyz* positive cells. (E) *gata1a* expression. Scale bar is shown in the Figure. *P < 0.05, **P < 0.01, ***P < 0.001.

**Supplemental Figure 4. Expression of EGFP and gbp4 fusion.** All components were injected with 200 pg RNA at the 1 cell stage and imaged with a fluorescent microscope at 10 hpf. Scale bar, 2 mm.

**Supplemental Figure 5. miRNA-7145 over-expression combined with cuedc2 over-expression partially restores blood development.** The embryos were injected with 200 pg miRNA-7145 mimics or co-injected 200 pg miRNA-7145 mimics and 200 pg cuedc2 mRNA at the 1 cell stage, and the embryos were collected at 26 hpf. (A-D) Detection of myeloid cell marker expression at 26 hpf with whole mount in situ hybridization. (A) *mfap4* expression, (C) *lyz* expression. (B) Statistics of *mfap4* positive cells and (D) Statistics of *lyz* positive cells. (E) *gata1a* expression. Scale bar is shown in the Figure. *P < 0.05, **P < 0.01, ***P < 0.001.

**Supplemental Figure 6. miRNA-7145 knock-down combined with cuedc2 knock-down rescues the blood development.** The embryos were injected with 10 ng miRNA-7145 MO or co-injected 10 ng miRNA-7145 MO and 10 ng cuedc2 MO at the 1 cell stage, and the embryos were collected at 26 hpf. (A-B) Detection of *lyz* expression at 26 hpf with whole mount *in situ* hybridization. (A) *lyz* expression. (B) Statistics of *lyz* positive cells. (C) *gata1a* expression. Scale bar is shown in the Figure. *P < 0.05, **P < 0.01, ***P < 0.001.

**Supplemental Figure 7. Hematopoiesis regulation model of miRNA-7145.** Over-expression of miRNA-7145 can inhibit the expression of cuedc2, thus inhibiting myeloid cell differentiation and promoting erythropoiesis. Knockdown of miRNA-7145 can promote the expression of cuedc2, thus promoting myeloid cell differentiation and inhibiting erythrogenesis. Cuedc2 can inhibit the JAK1/STAT3 signaling pathway which has been reported to contribute to erythropoiesis.

**Supplemental Figure 8. EGFP-cuedc2 sequence for *in vitro* transcription.** Green represents the EGFP sequence, red represents the CDS of cuedc2, and orange represents the 3’ UTR of cuedc2.
