# Supplementary figures and images for: miRNA-7145-cuedc2 axis controls hematopoiesis through JAK1/STAT3 signaling pathway
Source: Cell Death Discov. 2024 May 2;10:209. doi: 10.1038/s41420-024-01977-6 (PMC11066045; doi:10.1038/s41420-024-01977-6)

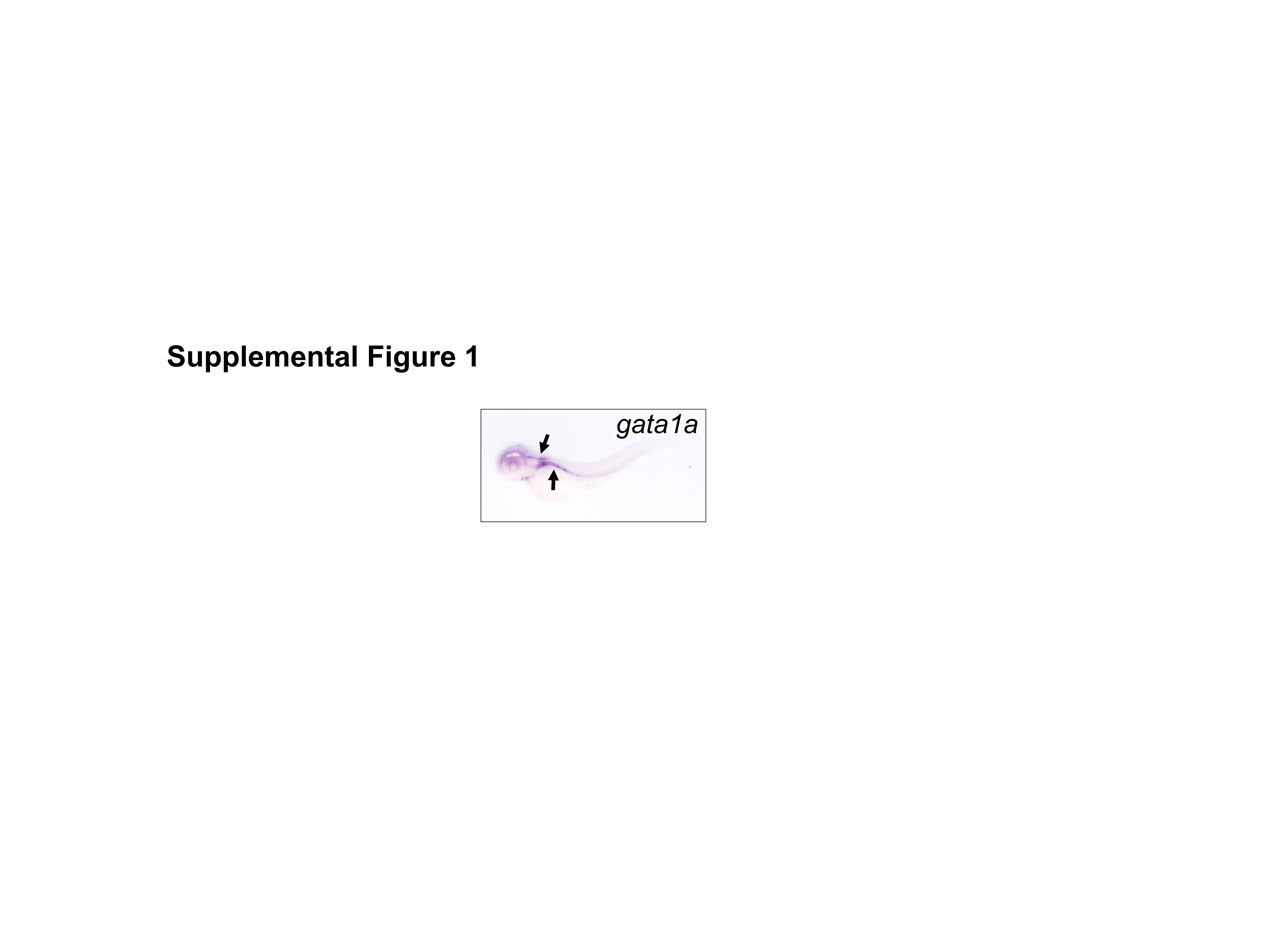

Supplement: Supplementary file 2 — Supplemental Figure 1 [file 41420_2024_1977_MOESM2_ESM.jpg]

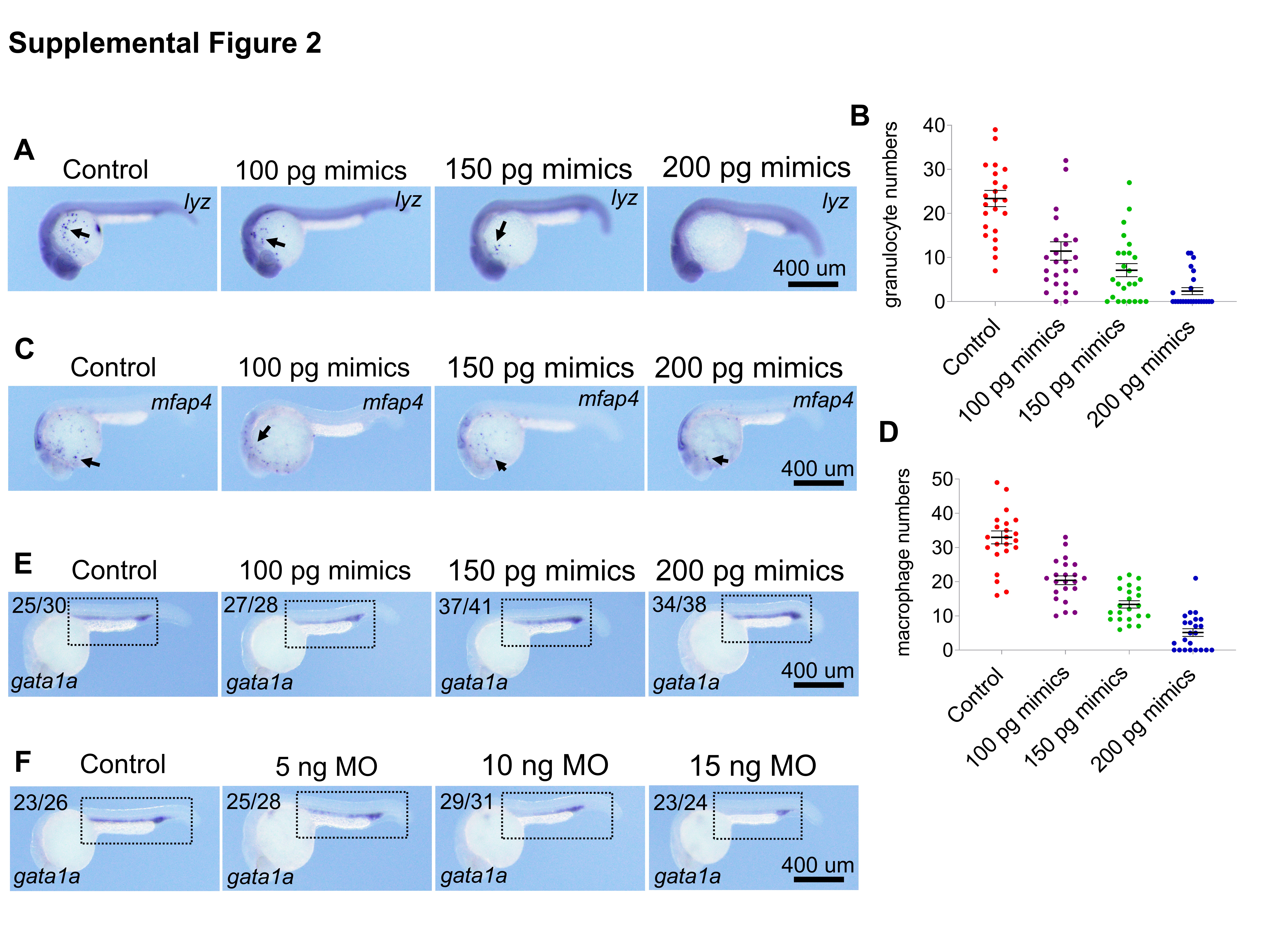

Supplement: Supplementary file 3 — Supplemental Figure 2 [file 41420_2024_1977_MOESM3_ESM.jpg]

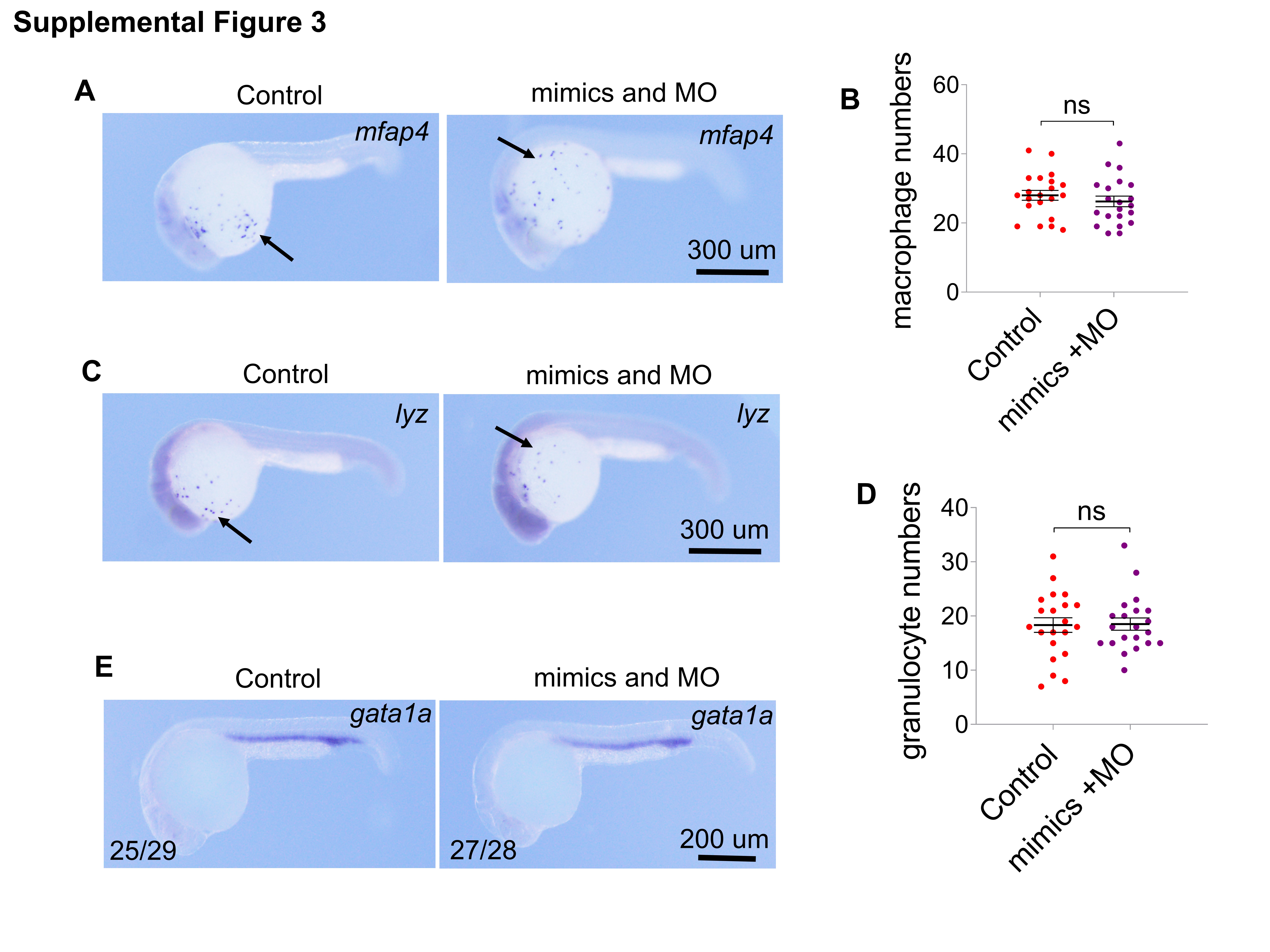

Supplement: Supplementary file 4 — Supplemental Figure 3 [file 41420_2024_1977_MOESM4_ESM.jpg]

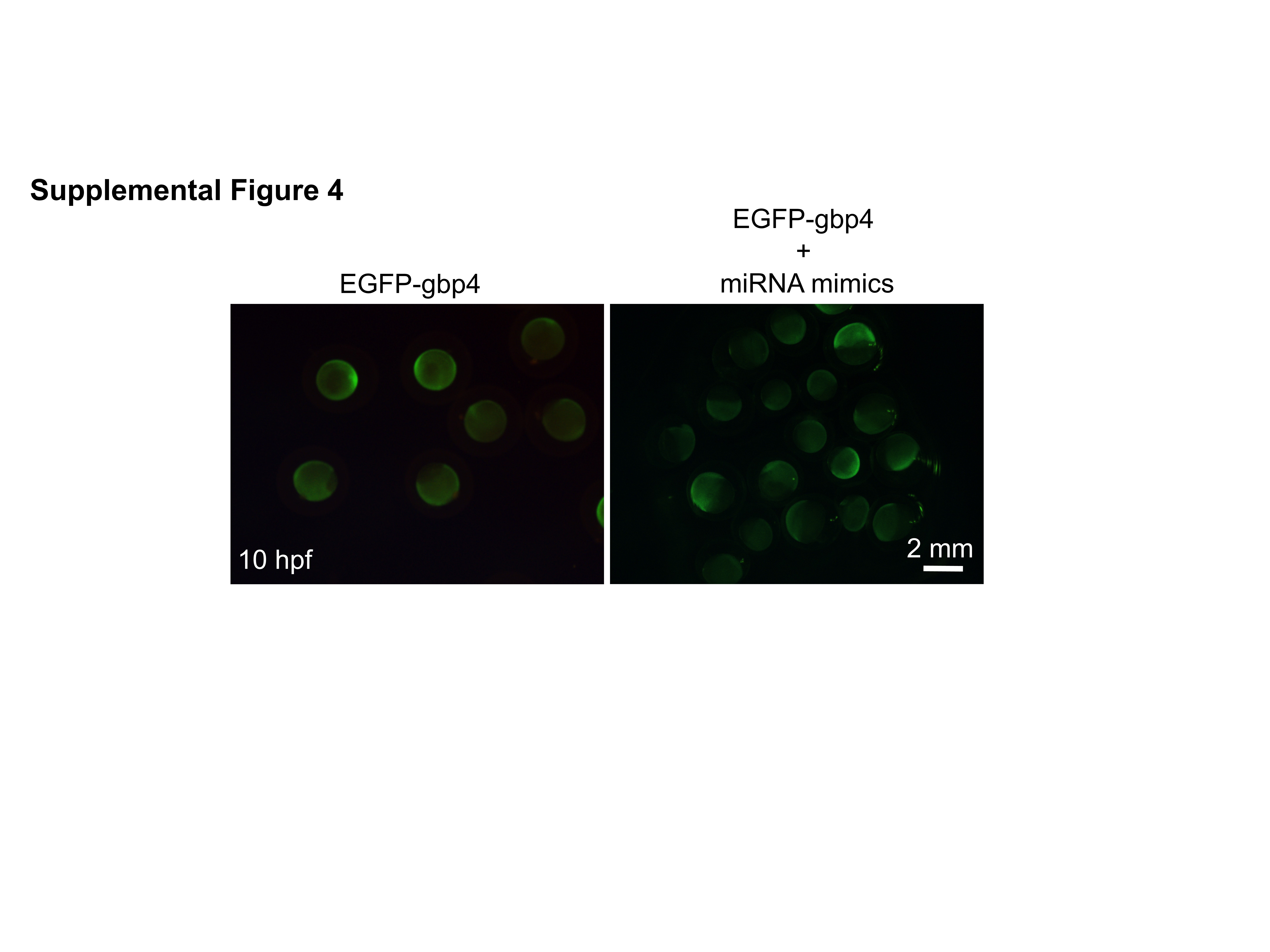

Supplement: Supplementary file 5 — Supplemental Figure 4 [file 41420_2024_1977_MOESM5_ESM.jpg]

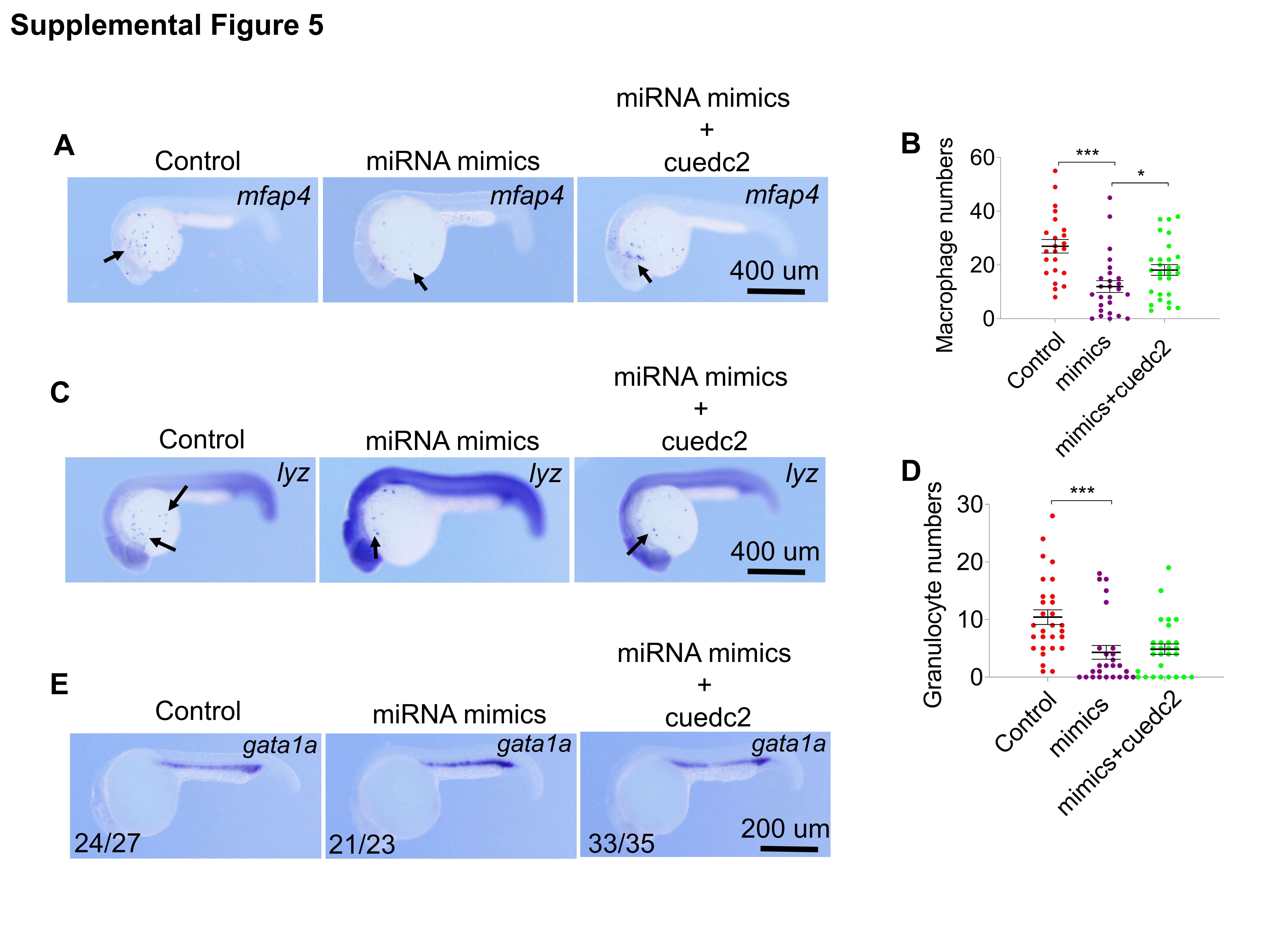

Supplement: Supplementary file 6 — Supplemental Figure 5 [file 41420_2024_1977_MOESM6_ESM.jpg]

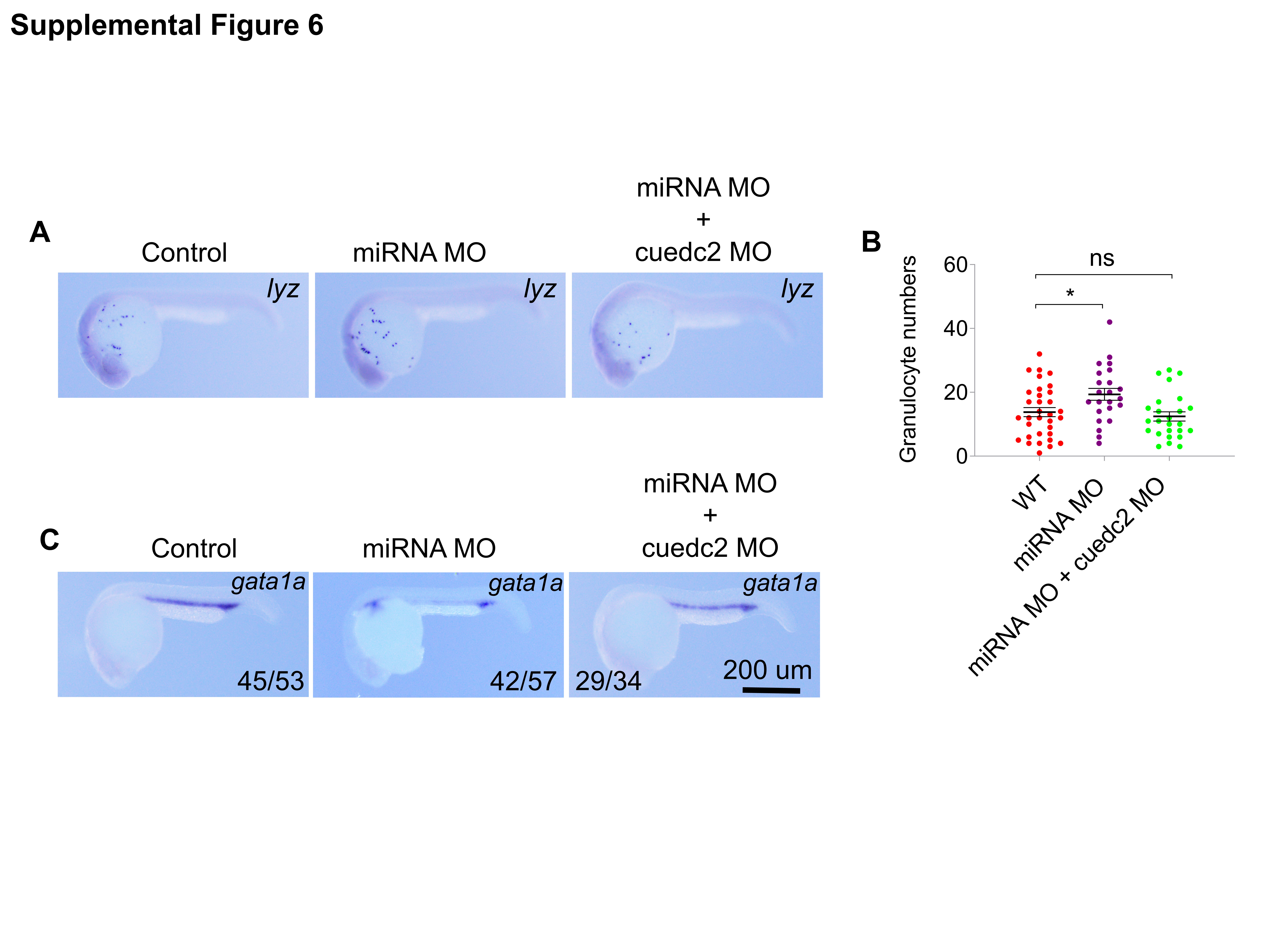

Supplement: Supplementary file 7 — Supplemental Figure 6 [file 41420_2024_1977_MOESM7_ESM.jpg]

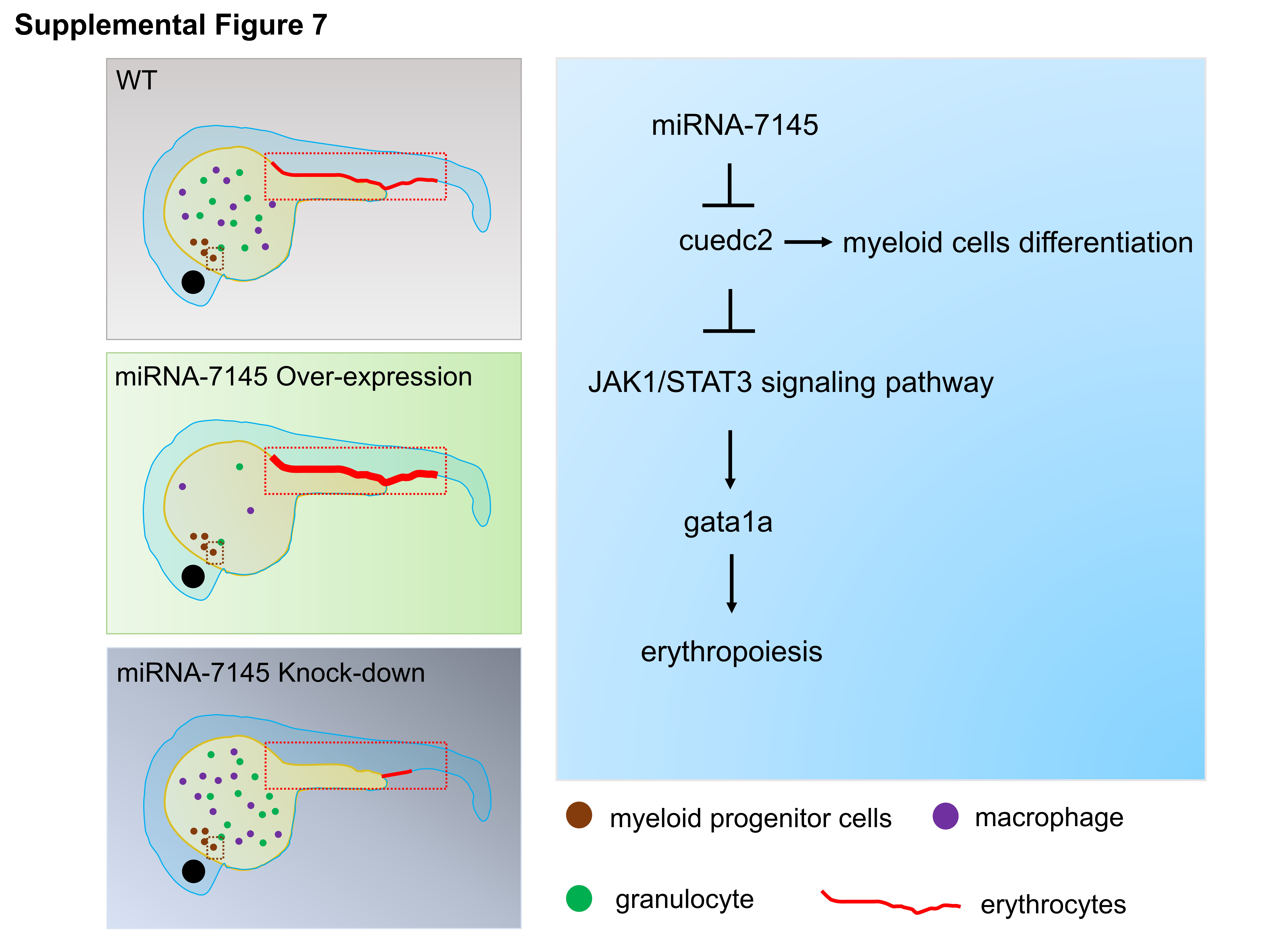

Supplement: Supplementary file 8 — Supplemental Figure 7 [file 41420_2024_1977_MOESM8_ESM.jpg]

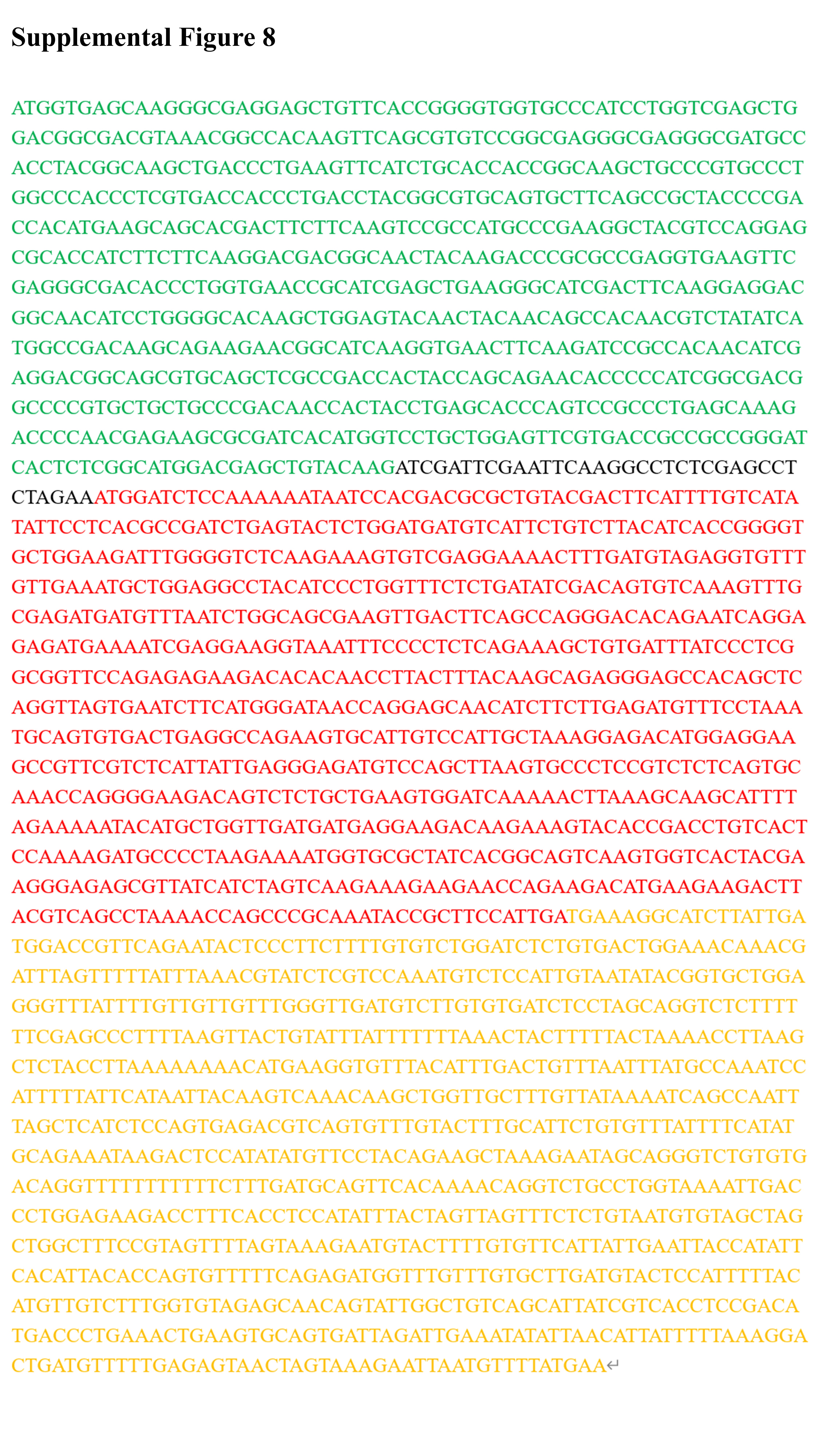

Supplement: Supplementary file 9 — Supplemental Figure 8 [file 41420_2024_1977_MOESM9_ESM.jpg]

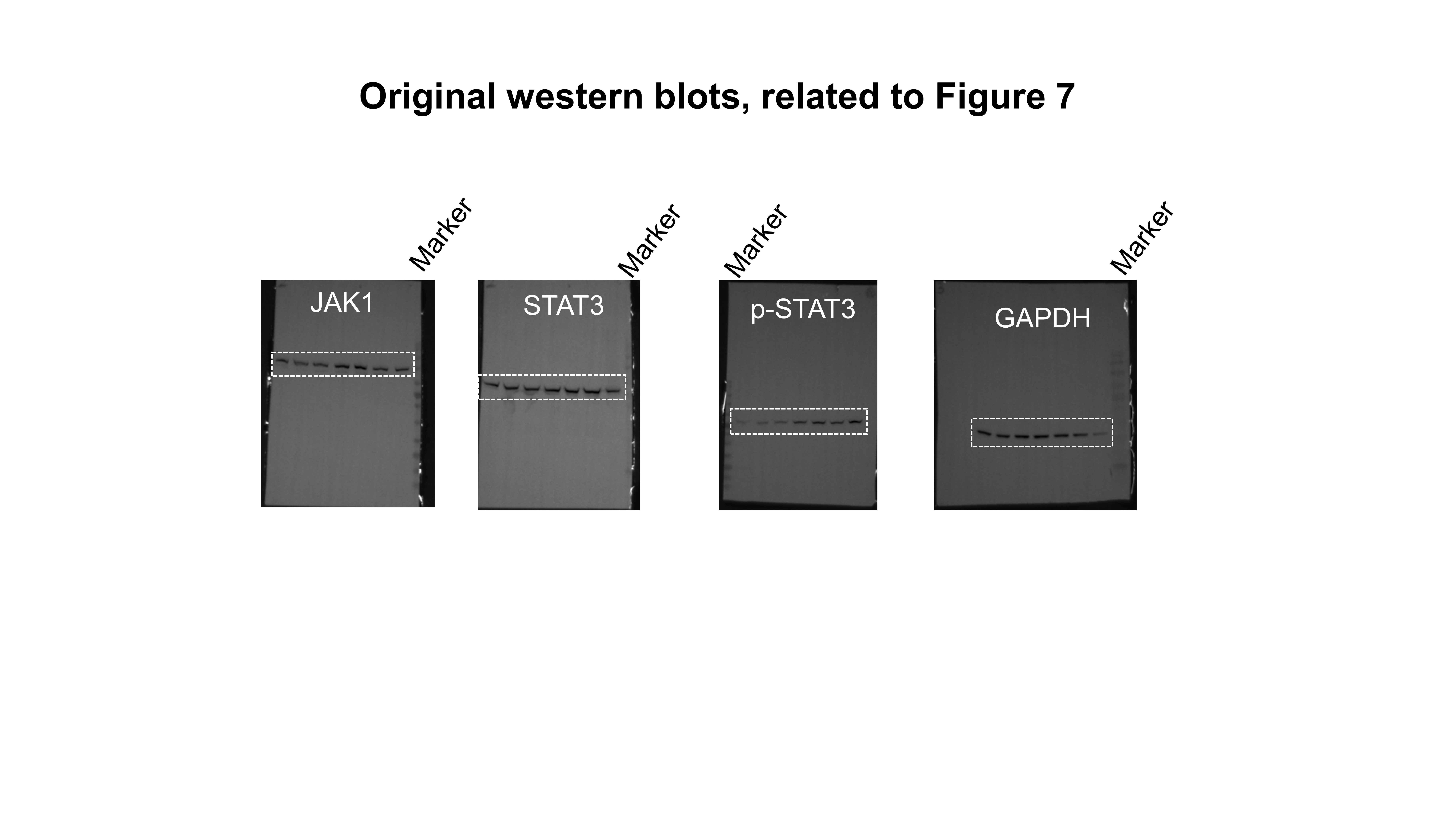

Supplement: Supplementary file 10 — WB original data [file 41420_2024_1977_MOESM10_ESM.jpg]
